# Variation in HIV-1 R5 macrophage-tropism correlates with sensitivity to reagents that block envelope: CD4 interactions but not with sensitivity to other entry inhibitors

**DOI:** 10.1186/1742-4690-5-5

**Published:** 2008-01-18

**Authors:** Paul J Peters, Maria J Duenas-Decamp, W Matthew Sullivan, Richard Brown, Chiambah Ankghuambom, Katherine Luzuriaga, James Robinson, Dennis R Burton, Jeanne Bell, Peter Simmonds, Jonathan Ball, Paul R Clapham

**Affiliations:** 1 Center for AIDS Research, Program in Molecular Medicine and Department of Molecular Genetics and Microbiology, 373 Plantation Street, University of Massachusetts Medical School, Worcester, MA 01605, USA; 2 Microbiology and Infectious Diseases, Institute of Infection, Immunity and Inflammation, The University of Nottingham, Queen's Medical Centre, Nottingham NG7 2UH, UK; 3 Center for AIDS Research, Program in Molecular Medicine and Department of Pediatrics, 373 Plantation Street, University of Massachusetts Medical School, Worcester, MA 01605, USA; 4 Department of Pediatrics, Tulane University School of Medicine, 1430 Tulane Avenue, New Orleans, LA 70112, USA; 5 The Scripps Research Institute, Departments of Immunology and Molecular Biology, IMM2, La Jolla, CA 92037, USA; 6 Department of Neuropathology, Western General Hospital, Crewe Road, Edinburgh, EH4 2XU, UK; 7 Centre for Infectious Diseases, University of Edinburgh, Summerhall, Edinburgh, EH9 1QH, UK

## Abstract

**Background:**

HIV-1 R5 viruses cause most of the AIDS cases worldwide and are preferentially transmitted compared to CXCR4-using viruses. Furthermore, R5 viruses vary extensively in capacity to infect macrophages and highly macrophage-tropic variants are frequently identified in the brains of patients with dementia. Here, we investigated the sensitivity of R5 envelopes to a range of inhibitors and antibodies that block HIV entry. We studied a large panel of R5 envelopes, derived by PCR amplification without culture from brain, lymph node, blood and semen. These R5 envelopes conferred a wide range of macrophage tropism and included highly macrophage-tropic variants from brain and non-macrophage-tropic variants from lymph node.

**Results:**

R5 macrophage-tropism correlated with sensitivity to inhibition by reagents that inhibited gp120:CD4 interactions. Thus, increasing macrophage-tropism was associated with increased sensitivity to soluble CD4 and to IgG-CD4 (PRO 542), but with increased resistance to the anti-CD4 monoclonal antibody (mab), Q4120. These observations were highly significant and are consistent with an increased affinity of envelope for CD4 for macrophage-tropic envelopes. No overall correlations were noted between R5 macrophage-tropism and sensitivity to CCR5 antagonists or to gp41 specific reagents. Intriguingly, there was a relationship between increasing macrophage-tropism and increased sensitivity to the CD4 binding site mab, b12, but decreased sensitivity to 2G12, a mab that binds a glycan complex on gp120.

**Conclusion:**

Variation in R5 macrophage-tropism is caused by envelope variation that predominantly influences sensitivity to reagents that block gp120:CD4 interactions. Such variation has important implications for therapy using viral entry inhibitors and for the design of envelope antigens for vaccines.

## Introduction

HIV-1 infection is triggered by interactions between the viral envelope glycoprotein and cell surface receptor CD4 and either of the coreceptors; CCR5 or CXCR4. These interactions induce the fusion of viral and cellular membranes and viral entry into cells. CCR5-using (R5) viruses are mainly transmitted [1], while CXCR4-using (X4) variants can be isolated from up to 50% of AIDS patients in subtype B infections and correlate with a more rapid loss of CD4^+ ^T-cells and faster disease progression [2-5]. Among T-cells, CCR5 expression is mainly restricted to memory T-cells [6,7], while CXCR4 is more widely expressed on various CD4^+ ^T-cell populations including naïve T-cells [6]. R5 viruses therefore target CCR5^+ ^memory T-cell populations and in the acute phase of replication, decimate the populations of CD4^+ ^memory cells in lymphoid tissue associated with the gut and other mucosa [8-10]. CCR5 is also expressed on macrophage lineage cells [7] in non-lymphoid tissues e.g. the brain [11], and R5 viruses predominantly target these cells in neural tissues [12-14]. When CXCR4-using viruses emerge in late disease, they colonize naïve T-cell populations that were not infected by R5 viruses [15,16]. Nonetheless, CD4 depletion and AIDS occur in patients from which only CCR5-using viruses can be isolated [17,18]. In clade C infections, CXCR4-using variants have been detected in far fewer individuals in the late stages of disease [17,19-22]. Thus, AIDS and death presumably occurs in the absence of CXCR4-using variants for a substantial number of HIV^+ ^patients and is caused directly by R5 viruses.

R5 viruses are frequently regarded as macrophage-tropic. However, several groups have reported considerable variation in the cell tropism of R5 viruses [23-25]. We reported that primary HIV-1 R5 isolates varied in their capacity to infect primary macrophage cultures by over 1000-fold [25] and we first described a subset of HIV-1 R5 isolates that could infect CD4^+ ^T-cell lines via trace amounts of CCR5 [23]. More recently, we described R5 envelopes amplified from brain and lymph node tissue of AIDS patients that also differed markedly in tropism properties [26,27]. Thus R5 envelopes from brain tissue were highly macrophage-tropic and were able to exploit low amounts of CD4 and/or CCR5 for infection. They contrasted considerably with R5 envelopes from immune tissue (lymph node) that conferred inefficient macrophage infection and required high amounts of CD4 for infection. Moreover, these non-macrophage-tropic envelopes were more prevalent (than macrophage-tropic envelopes) amplified from immune tissue, blood or semen [27]. These results generally support earlier reports that described a small number of highly macrophage-tropic R5 virus isolates made from brain tissue [28]. Others have confirmed that envelopes amplified from brain tissue can infect cells via low CD4 levels [29,30]. However, Thomas et al. reported less compartmentalized variation of R5 macrophage tropism, with macrophage-tropic R5 envelopes present in both lymphoid and brain tissue [30]. The capacity of highly macrophage-tropic envelopes to use low amounts of CD4 and/or CCR5 suggests that such variants could also confer a broader tropism among CD4^+ ^T-cells (that express low amounts of these receptors) and contribute to CD4^+ ^T-cell depletion late in disease if they are present in immune tissue.

Several groups have also reported differences in the properties of R5 virus isolates made from blood. Thus, virus isolates from late disease were reported to be more macrophage-tropic than those from earlier stages [31-33]. In addition, Repits et al. described late disease isolates with increased replicative capacity and reduced sensitivity to entry inhibitors including TAK779 (CCR5 antagonist) and T20 (gp41 inhibitor) [34]. However, they did not test whether these late isolates could exploit low CD4 or infect macrophages. It is unclear whether the highly macrophage-tropic envelopes that we have amplified from brain tissue and other sites, correspond to the late isolates described by other groups [31-34].

Recently, Dunfee et al. described a polymorphism in the C2 region of the CD4 binding site on gp120. Thus, 41% of their envelope sequences from brain tissue of patients with dementia carried an asparagine at residue 283 compared with 8% of envelopes from patients without dementia [35]. We also reported a predominance of N283 in highly macrophage-tropic brain envelopes compared to lymph node, blood and semen [27]. N283 was shown to increase the affinity of monomeric gp120 for CD4 [35]. More recently, the loss of a glycosylation site (N386) close to the CD4 binding loop on gp120 was reported to occur more frequently in HIV in the brain and was shown to contribute to increased R5 macrophage-tropism [36], an observation that we have recently confirmed (Duenas-Decamp et al. Personal communication).

How variation in R5 tropism impacts on the sensitivity of HIV-1 to neutralizing antibodies and entry inhibitors is unclear. We, and others have reported that R5 macrophage-tropism correlated with increased resistance to anti-CD4 monoclonal antibodies (mabs), consistent with an increased affinity between gp120 and CD4. However, there was no correlation with sensitivity to the CCR5 antagonist, TAK779 [26,29]. Here, we have extensively analyzed the sensitivity of thirty-six envelopes from brain, LN, blood and semen to a range of reagents that block HIV-1 entry. All these envelopes were derived from patient material by PCR without culture and have therefore not been altered by viral isolation procedures. Reagents tested for inhibition included soluble CD4 (sCD4) and tetrameric IgG-CD4 (PRO 542), BMS-378806; a small molecule that targets a site deep in the cleft that binds CD4, mabs to CD4 and CCR5, CCR5 antagonists, T20 and human mabs that recognize conserved neutralization epitopes on gp120 and gp41.

Our results strongly suggest that R5 macrophage-tropism is primarily modulated by changes in the CD4 binding site on gp120 and in its affinity for CD4. Such changes impact on sensitivity to the CD4bs mab, b12 and may be driven by the presence or absence of neutralizing antibodies *in vivo *that target the CD4bs or proximal sites. If highly macrophage-tropic R5 variants are preferentially transmitted, then vaccines that generate antibodies to the CD4bs may be particularly effective at preventing viral transmission.

## Results

### Macrophage-tropism of brain and lymph node envelopes

Envelopes used here have been described previously [26,27] with the addition of SQ43 380.4. They are all R5, predominantly using CCR5 as a coreceptor [26,27]. Table 1 shows macrophage infectivity as a percentage of the titer recorded on HeLa TZM-BL cells as described previously [27]. Macrophage infectivity was highly variable. Envelopes that conferred macrophage infectivity of >0.5% of infectivity for HeLa TZM-BL cells were designated as macrophage-tropic and are shown by bold script in Table 1. This arbitrary designation allows for easy identification of these envelopes as grey symbols in subsequent figures. All but one brain envelope conferred macrophage infection. None of the env^+ ^pseudovirions carrying lymph node envelopes conferred significant macrophage infection. Macrophage-tropic R5 envelopes were amplified less frequently from blood and semen of adults and in plasma of infants.

**Table 1:** Macrophage tropism of R5 envelopes studied.

Patient Number	Envelope	Macrophage Infectivity (%)^a^	Patient Number	Envelope	Macrophage Infectivity (%)^a^
NA20	B59	**16.9**^b^	P1114	C95-65	0.029
	B76	**0.179**		C96-26	0.097
	B501	**51.6**		C98-15	**32.4**
	LN3	<0.001		C98-18	**2.21**
	LN8	<0.001		C98-27	0.144
	LN10	0.030		C98-28	0.004
	LN14	0.025		C98-67	0.003
	LN16	0.036	P3	Q3 164 1.4	0.002
NA420	B13	**0.335**		Q3 180 6.4	0.003
	B33	**3.35**		SQ3 196 10.1	0.012
	B42	**0.559**		SQ3 197 9.3	**0.338**
	LN40	0.009		SQ3 199 8.5	0.003
	LN85	0.026	P31	Q31 350.1	0.05
NA118	B12	0.006		Q31 351.6	0.02
	LN27	0.023		SQ31 308.2	0.02
	LN33	0.023	P43	Q43 378.2	0.03
NA176	B93	**8.2**		SQ43 380.1	**0.6**
NA353	B27	**12.6**		SQ43 380.4	**9.63**
					
			Controls	AD8	**4.60**
				SF162	**6.25**
				YU2	**6.36**
				JRFL	**3.27**
				JRCSF	0.011

^a^Macrophage infectivity as a percent of infectivity recorded on HeLa TZM-BL cells. Most of this data is derived from that presented in Peters et al. [27] with the addition of SQ43 380.4.

^b^Bolded percentages indicate envelopes that were designated as macrophage-tropic i.e. >0.5% of infectivity for HeLa TZM-BL cells.

### The effect of variation in R5 envelope tropism on sensitivity to entry inhibitors and neutralizing antibodies

In immune tissue where there are high levels of neutralizing antibodies, the HIV-1 envelope may evolve to protect critical sites (e.g. the CD4bs) from antibodies. In contrast, the brain is enclosed by the blood brain barrier, which usually restricts immunoglobulin from entering [37,38]. HIV-1 variants replicating in the brain may therefore evolve stronger interactions with CD4 and/or CCR5 resulting from enhanced exposure of the CD4 and/or CCR5 binding sites, but become more vulnerable to antibody neutralization. We tested the sensitivity of our panel of brain, LN, blood and semen envelopes to a range of entry inhibitors and monoclonal antibodies. The entry inhibitors specifically block interactions of envelope with CD4 or CCR5, or prevent gp41 conformational changes required for fusion, while monoclonal antibodies sterically inhibit infection by binding conserved envelope sites on virions.

### Inhibitors and antibodies that interfere with envelope:CD4 interactions

**Figure 1:**
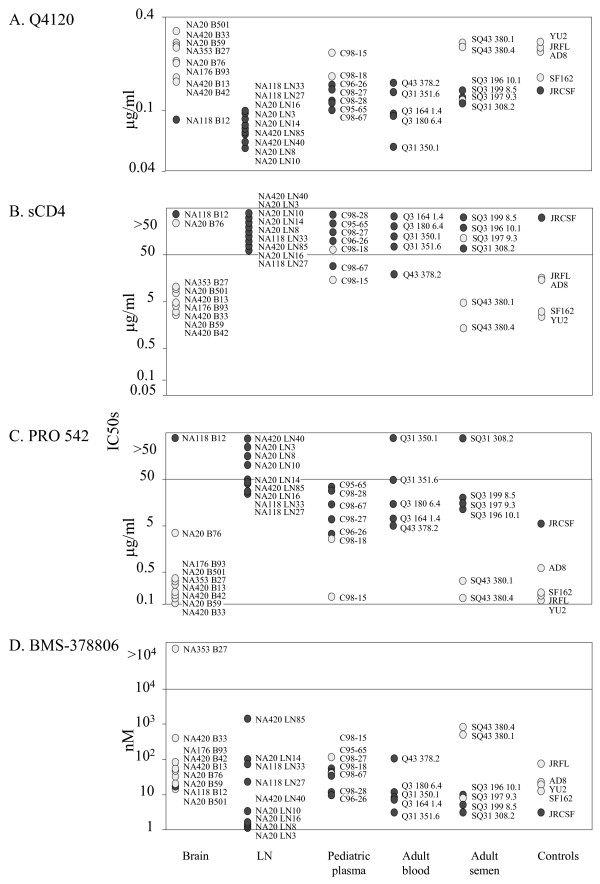
Sensitivity of HIV-1 R5 envelopes to reagents that interfere with gp120:CD4 interactions. Pseudovirions carrying envelopes encoded by envelope genes amplified from patient samples were tested for sensitivity to inhibition by (A) anti-CD4 mab, Q4120, (B) sCD4, (C) PRO 542 and (D) BMS-378806. Macrophage-tropic envelopes (light symbols) were more sensitive to sCD4 and PRO 542 compared to non-macrophage-tropic envelopes (dark symbols) but were more resistant to the anti-CD4 mab, Q4120.

Figure 1A shows that macrophage-tropic envelopes were more resistant to inhibition by the CD4 mab, Q4120, which binds domain 1 of CD4 and competes with envelope for binding to CD4. In contrast, the same macrophage-tropic envelopes were more sensitive to soluble CD4 (sCD4) (Figure 1B) and to the more potent tetrameric IgG-CD4 construct (PRO 542) (Figure 1C). We used two-tailed non-parametric Spearman analyses to evaluate whether macrophage-tropism correlated with sensitivity to these reagents. Importantly, such analyses do not rely on our arbitrary designation of macrophage-tropism but simply compare macrophage infectivity titers (Table 1) with IC50s for each inhibitor. Our results showed highly significant correlations between increasing macrophage-tropism and increased sensitivity to sCD4 and PRO 542 as well as with an increased resistance to Q4120 (Table 2). These results are consistent with an increased affinity of R5 macrophage-tropic gp120s for binding to CD4, although alternative explanations should also be considered (see below). Statistical evaluations of correlations between R5 macrophage-tropism and sensitivity to different inhibitors are discussed more fully below and p values are shown in Table 2.

**Table 2:** Non-parametric two-tailed Spearman analysis for correlations between R5 envelope macrophage-tropism and sensitivity to entry inhibitors.

Inhibitor/Antibody	Target of reagent	Stage of entry blocked^3^.	Correlation with Macrophage-tropism (p Values)
Q4120	CD4	env: CD4 interactions	<0.0001**
sCD4	gp120, CD4bs	env: CD4 interactions	<0.0001**
PRO 542 (IgG-CD4)	gp120, CD4bs	env: CD4 interactions	<0.0001**
BMS-378806	gp120, CD4bs channel^1^.	env: CD4 interactions	0.0002**
b12	gp120, overlapping CD4bs^2^.		0.6843
TAK779	CCR5	env: CCR5 interactions	0.7964
SCH350581	CCR5	env: CCR5 interactions	0.7587
2D7	CCR5	env: CCR5 interactions	
2G12	gp120 glycan	env: CCR5 interactions	0.0138*
T20	gp41 conformational changes	gp41 conformational changes	0.7061
2F5	gp41 membrane proximal region	gp41 conformational changes^4^.	0.3741
4E10	gp41 membrane proximal region	gp41 conformational changes^4^.	0.3502

1. BMS-378806 binds to a hydrophobic channel deep in the channel targeted by CD4 [42].

2. Mab b12 binds an epitope that overlaps the CD4bs [57].

3. Mab 2G12 binds to a glycan on gp120. 2G12 blocks env:CCR5 interactions but may also block earlier stages of entry [73].

4. Mabs 2F5 and 4E10 block gp41 conformational changes but may also block earlier stages of entry [73].

* Significant (p ≤ 0.05).

** Highly significant (p ≤ 0.01)

We also tested the small molecule, BMS-378806, which was reported to inhibit gp120 binding to CD4 [39-41] and subsequent conformational changes [42]. BMS-378806 is believed to bind into a deep hydrophobic channel of unliganded gp120 close to and underneath the sites that bind to CD4. Thus, BMS-378806 may directly inhibit CD4 binding and also act to stabilize the unliganded form of the gp120 [43]. There was also a highly significant correlation between R5 macrophage-tropism and BMS-378806 sensitivity (Table 2, see below). However, in contrast to sCD4 and tetrameric IgG-CD4, BMS-378806 sensitivity decreased with increasing macrophage-tropism.

**Figure 2:**
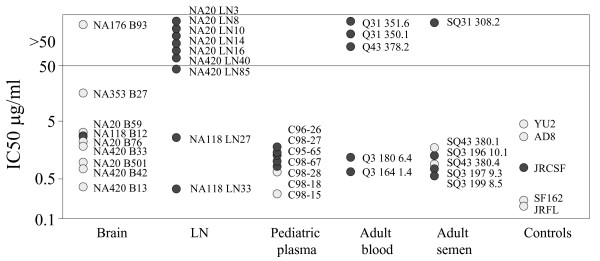
Sensitivity of HIV-1 R5 envelopes to the CD4bs mab, b12. Pseudovirions carrying envelopes encoded by envelope genes amplified from patient samples were tested for sensitivity to inhibition by b12. All but one of the macrophage-tropic envelopes (light symbols) were sensitive to b12, while many non-macrophage-tropic envelopes (dark symbols) were resistant.

We next tested envelope sensitivity to the CD4bs mab, b12 (Figure 2). All but one macrophage-tropic env conferred sensitivity to b12 neutralization, while many non-macrophage-tropic envelopes were resistant at 50 μg/ml antibody. These results indicate that there is also a strong relationship between b12 sensitivity and R5 envelope tropism, although this did not result in a statistically significant overall correlation (Table 2).

### Sensitivity of R5 envelopes to reagents that target envelope:CCR5 interactions

The mouse mab 17b binds to a conserved CD4-induced epitope on gp120 that overlaps the conserved part of the coreceptor binding site (not shown). None of the patient envelopes were inhibited by 17b, suggesting that this site is not more exposed on macrophage-tropic envelopes. However, 17b did neutralize T-cell line adapted HIV-1 isolates NL4.3 and HXBc2 (not shown).

**Figure 3:**
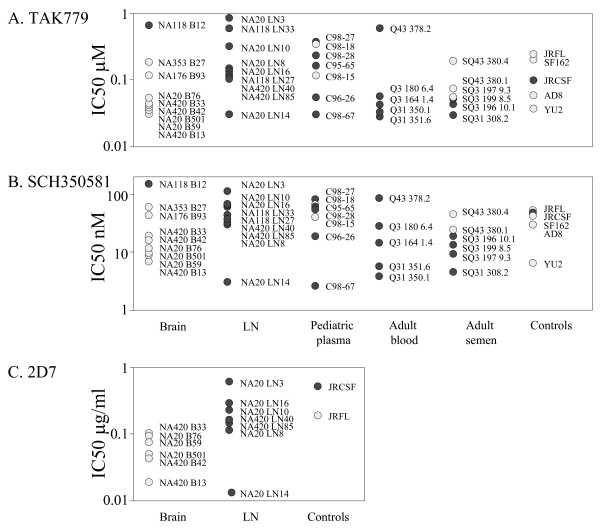
Sensitivity of HIV-1 R5 envelopes to reagents that interfere with gp120:CCR5 interactions. Pseudovirions carrying envelopes encoded by envelope genes amplified from patient samples were tested for sensitivity to inhibition by (A) TAK779, (B) SCH350581 and (C) anti-CCR5, 2D7. Macrophage-tropic envelopes (light symbols) and non-macrophage-tropic envelopes (dark symbols) were examined. Statistical analysis showed no overall correlation between macrophage-tropism and sensitivity to TAK779 or SCH350581 (Table 2).

In contrast, both CCR5 antagonists TAK779 and SCH350581 inhibited all the envelopes regardless of their tropism for macrophages (Figures 3A and 3B). As expected SCH350581 was a substantially more potent inhibitor compared to TAK779. In contrast to the strong correlations observed between macrophage-tropism and reagents that inhibited gp120:CD4 interactions, overall correlations with sensitivity to CCR5 antagonists were not significant (Table 2). CCR5 antagonists bind to a cavity in between the transmembrane domains of CCR5. It is believed that these reagents confer a CCR5 structure that is no longer recognized by the HIV envelope [44,45]. Thus, although CCR5 antagonists compete with HIV for binding CCR5, they are not competing for the same site. It was possible that CCR5-specific inhibitors that compete directly with HIV for binding the extracellular regions of CCR5 may confer a different pattern of envelope sensitivity. We therefore tested the anti-CCR5 monoclonal antibody, 2D7, which binds ECL2 of CCR5, a region that interacts with sites on the V3 loop of envelope. Due to limiting amounts of 2D7, we tested only brain and LN envelopes from patients NA420 and NA20, with JRFL and JRCSF as controls. Figure 3C shows a trend of brain macrophage-tropic envelopes being more sensitive to 2D7 compared to LN envelopes, although this did not reach statistical significance (p = 0.0839). NA20 LN14 was a clear 'outlier' from other LN envelopes and was among the envelopes most sensitive to all three CCR5 inhibitors (see discussion below).

### Inhibition by human mab, 2G12 that targets gp120 glycosylation groups

**Figure 4:**
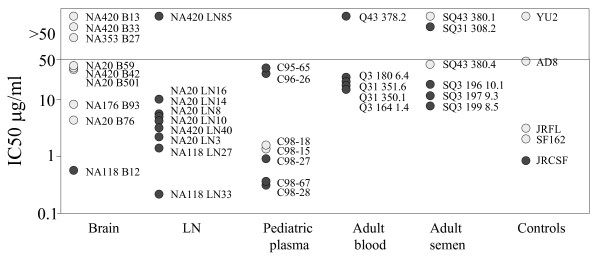
Sensitivity of HIV-1 R5 envelopes to 2G12. Pseudovirions carrying envelopes encoded by envelope genes amplified from patient samples were tested for sensitivity to inhibition by 2G12. Macrophage-tropic envelopes (light symbols) and non-macrophage-tropic envelopes (dark symbols) were examined. Statistical analysis showed a significant correlation between macrophage-tropism and sensitivity to 2G12.

The human monoclonal antibody, 2G12, neutralizes HIV-1 isolates mainly from clade B via relatively conserved glycosylation structures on gp120 [46,47]. Clear variation in sensitivity to 2G12 was noted, with most envelopes sensitive, while some were resistant (Figure 4). Of note, several brain-derived envelopes were resistant including NA420 envelopes B13, B33 and B42 as well as NA353 B27 and YU2. A significant correlation between macrophage-tropism and decreased 2G12 sensitivity was noted. Table 3 lists the presence or absence of glycosylation sites previously reported to be important for 2G12 binding [46,47]. All five of the NA420 envelopes lacked the critical potential glycosylation site at N339, while B13 and B33 also lacked N386. The loss of these glycosylation sites likely contributes to 2G12 resistance for some of these envelopes. However, LN40 is sensitive to 2G12 despite lacking N339, and NA353 B27 is resistant even though all the 2G12-implicated glycosylation sites are present. The determinants for 2G12 resistance and sensitivity for these envelopes are therefore unclear and will require further investigation to define precisely.

**Table 3:** R5 envelopes sensitivity to 2G12 neutralization and conservation of critical potential N-linked glycosylation sites.

Envelope		2G12 sensitivity	N295	N332	N339	N386	N392	N448
NA20	B59	+/-	+	+	+	+	+	+
	B76	+	+	+	+	+	+	+
	B501	+/-	+	+	+	+	+	+
	LN3	+	+	+	+	+	+	+
	LN8	+	+	+	+	+	+	+
	LN10	+	+	+	+	+	+	+
	LN14	+	+	+	+	+	+	+
	LN16	+	+	+	+	+	+	+

NA420	B13	-	+	+	-	-	+	+
	B33	-	+	+	-	-	+	+
	B42	+/-	+	+	-	+	+	+
	LN40	+	+	+	-	+	+	+
	LN85	-	+	+	-	+	+	+
								
NA118	B12	+	+	+	+	+	+	+
	LN27	+	+	+	+	+	+	+
	LN33	+	+	+	+	-	+	+
								
NA176	B93	+	+	+	+	+	+	+
								
NA353	B27	-	+	+	+	+	+	+
								
P-1114	C95-65	+/-	+	+	-	+	+	+
	C96-26	+/-	+	+	+	+	+	+
	C98-15	+	+	+	+	+	+	+
	C98-18	+	+	+	+	+	+	+
	C98-27	+	+	+	+	+	+	+
	C98-28	+	+	+	+	+	+	+
	C98-67	+	+	+	+	+	+	+
								
P3	Q3 164 1.4	+	+	+	+	+	+	+
	Q3 180 6.4	+/-	+	+	+	+	+	+
	SQ3 196 10.1	+	+	+	+	+	+	+
	SQ3 197 9.3	+	+	+	+	+	+	+
	SQ3 199 8.5	+	+	+	+	+	+	+
								
P31	Q31 350.1	+/-	+	+	+	+	+	+
	Q31 351.6	+/-	+	+	+	+	+	+
	SQ31 308.2	+/-	+	+	+	+	+	+
								
P43	Q43 378.2	-	+	+	+	+	+	-
	SQ43 380.1	-	+	+	+	+	+	-
	SQ43 380.4	-	+	+	+	+	+	-
								
Controls	AD8	+/-	+	+	+	+	+	+
	SF162	+	+	+	+	+	+	+
	YU2	-	+	+	-	+	+	+
	JRFL	+	+	+	+	+	+	+
	JRCSF	+	+	+	+	+	+	+

For 2G12 sensitivity; -, IC50 > 50 μg/ml; +/-, IC50 20–50 μg/ml; +, IC50 < 20 μg/ml.

### Inhibition by mabs 4E10 and 2F5 that bind membrane proximal epitopes on gp41

**Figure 5:**
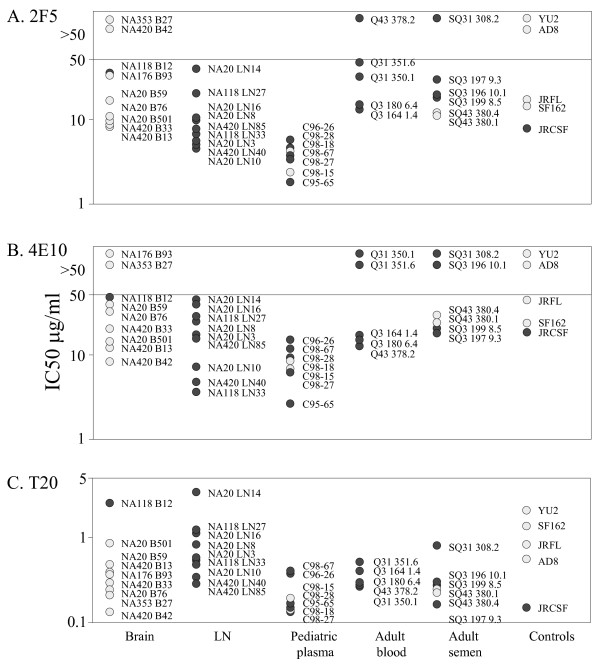
Sensitivity of HIV-1 R5 envelopes to reagents that target gp41 and inhibit conformational changes in gp41 required for fusion. Pseudovirions carrying envelopes encoded by envelope genes amplified from patient samples were tested for sensitivity to inhibition by (A) mab 2F5, (B) mab 4E10 and (C) T20. Macrophage-tropic envelopes (light symbols) and non-macrophage-tropic envelopes (dark symbols) were examined. Statistical analysis showed no overall correlation between macrophage-tropism and sensitivity to 2F5, 4E10 or T20. However, when just brain and lymph node envelopes were evaluated, a correlation between macrophage-tropism and increased sensitivity to T20 was nearly reached (p = 0.0658).

Figures 5A, 5B and Table 2 show that there was also no clear correlation between macrophage-tropism and sensitivity to the mabs 4E10 and 2F5 that bind conserved membrane proximal epitopes on gp41. Of the envelopes that conferred 2F5 resistance, only NA420 B42 (ELDNWA) did not contain the core ELDKWA epitope associated with 2F5 sensitivity [48-50].

### Inhibition by T20 that inhibits formation of the gp41 6-helix bundle required for fusion

All envelopes tested were sensitive to T20 (Figure 5C). However, no overall correlation was observed between T20 sensitivity and R5 macrophage-tropism. The envelope determinants of resistance and sensitivity to T20 shown here are unclear. All envelopes carried the GIV 36–38 motif in HR1, the site where resistance mutations frequently appear [51,52].

### Summary of correlations between macrophage-tropism and sensitivity to inhibitors

**Figure 6:**
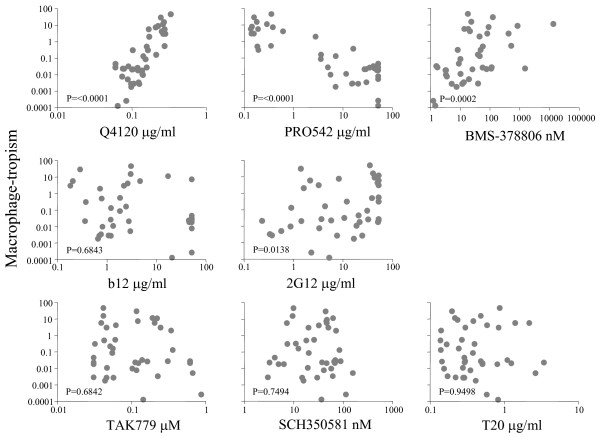
Sensitivity of HIV-1 R5 envelopes to inhibition by Q4120 and PRO 542 correlates with macrophage-tropism. HIV-1 R5 macrophage-tropism correlated with an increased sensitivity to PRO 542 but decreased sensitivity to Q4120 and BMS378806. HIV-1 R5 macrophage-tropism also correlated with sensitivity to 2G12 but not with b12, TAK779, SCH350581 or T20 (see complete list of p values in Table 2).

Table 2 and Figure 6 show that R5 macrophage-tropism correlates with sensitivity to inhibitors that interfere with gp120:CD4 interactions. There was also a significant correlation between increased macrophage-tropism and with decreased sensitivity to 2G12 neutralization. No overall correlation was noted between macrophage-tropism and sensitivity to the gp41 mabs or T20. In summary, R5 macrophage-tropism correlated with sensitivity to reagents that interfere with gp120:CD4 binding but not with inhibitors that prevent gp120, CCR5 interactions or gp41 conformational changes.

### Intrapatient variation in sensitivity to b12, and CCR5 antagonists

**Figure 7:**
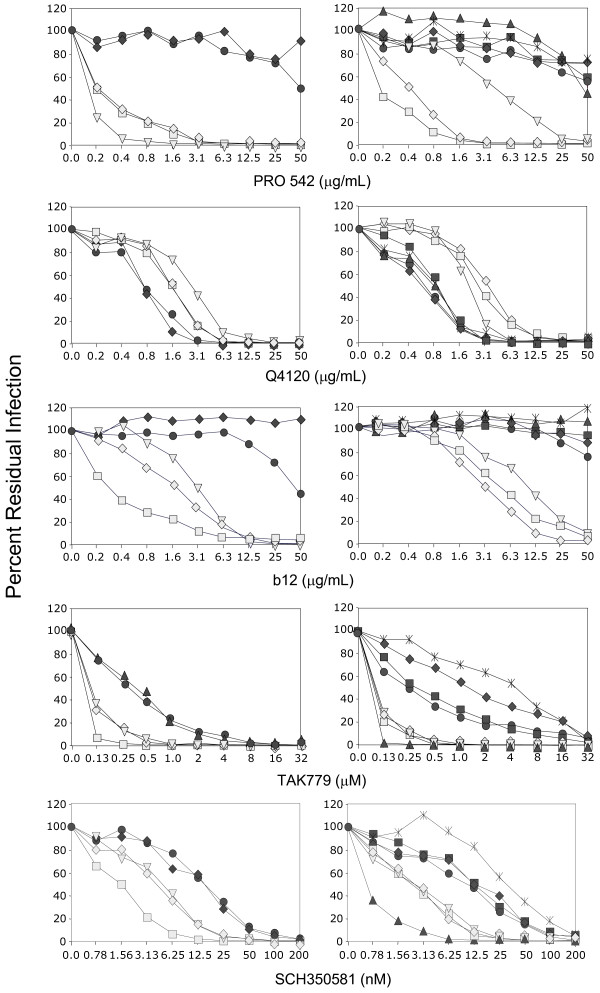
Intrapatient variation of HIV-1 R5 envelope macrophage-tropism and sensitivity to reagents that inhibit gp120 interactions with CD4 and CCR5. Brain-derived envelopes (light symbols) from patient NA420 (left panels) and NA20 (right panels) were more sensitive to PRO 542, b12, TAK779 and SCH350581, but more resistant to anti-CD4, Q4120, compared to LN-derived envelopes (dark symbols). NA420 envelopes tested were B13 (light squares), B33 (light triangles), B42 (light diamonds), LN85 (dark circles) and LN40 (dark triangles). NA20 envelopes tested were B59 (light squares), B76 (light triangles), B501 (light diamonds), LN3 (crosses), LN8 (dark circles), LN10 (dark diamonds), LN14 (dark triangles) and LN16 (dark squares).

Although all but one of the macrophage-tropic brain envelopes were sensitive to b12 and most non-macrophage-tropic envelopes were resistant, there was not a significant correlation between macrophage-tropism and b12 sensitivity. However, Figure 7 shows dose dependent b12 neutralization profiles for brain and lymph node envelopes from patients NA20 and NA420. For both patients, all macrophage-tropic brain envelopes were more sensitive to b12, while non-macrophage-tropic LN envelopes were resistant.

Figure 7 also shows dose dependent variation in TAK779 and SCH350581 for envelopes from patients NA20 and NA420. For both patients, the macrophage-tropic brain envelopes were more sensitive to TAK779 and SCH350581 compared to most or all of the non-macrophage-tropic LN envelopes. These results do not support an increase in envelope: CCR5 affinity for highly macrophage-tropic brain envelopes as suggested by an earlier study [28].

Together these results show clear intrapatient and tissue modulation of envelope sensitivity to b12 and to TAK779 and SCH350581. Similar tissue specific sensitivity was also observed for the NA20 and NA420 envelopes with PRO 542 and Q4120 (Figure 7), sCD4 (not shown), and 2D7 (Figure 3C).

## Discussion

For the majority of HIV^+ ^patients, AIDS and death result from replication by HIV-1 R5 viruses in the absence of detectable CXCR4-using variants. The mechanisms of CD4^+ ^T-cell loss and immune destruction conferred by R5 viruses are unclear. Whether R5 variants with increased virulence emerge in late disease and contribute to CD4^+ ^T-cell loss remains an open question. Several groups have reported the presence of R5 variants in late disease that are highly macrophage-tropic [31-33]. The capacity of highly macrophage-tropic R5 viruses to infect cells with low levels of CD4 and/or CCR5 may confer a broader tropism for CD4^+ ^T-cells and exacerbate their depletion late in disease. Our previous studies have highlighted the variation of R5 viruses at different tissue sites [26,27], showing that highly macrophage-tropic R5 envelopes predominated in brain tissue but were less prevalent in immune tissue (lymph node), blood and semen.

In this study we have examined the sensitivity of envelopes amplified from these different sites to a range of inhibitors and antibodies that target CD4, CCR5, or various sites on the HIV envelope and block different stages in the entry process. We focused entirely on R5 envelopes and did not include R5X4 or X4 envs. We evaluated whether the variation in macrophage-tropism estimated for all R5 envelopes correlated with sensitivity to each of these reagents using a two-tailed, non-parametric Spearman test with 95% confidence limits. Care must be taken in interpreting these analyses since the panel of envelopes evaluated included several sets that originated from individual subjects i.e. thirty-six envelopes from nine subjects. Thus, it is possible that envelopes with a particular phenotype may be predominant in an individual due to a founder effect or other extenuating circumstances and shift the statistical significance in its favor. Nonetheless, envelope sensitivity to reagents that block CD4: gp120 interactions (sCD4, IgG-CD4 and Q4120) correlated with R5 macrophage-tropism with very high significance. Thus, our data strongly indicates that R5 macrophage tropism predominantly correlates with sensitivity to reagents that interfere with envelope binding to CD4. Macrophage-tropic R5 viruses were more sensitive to sCD4 and tetravalent IgG-CD4 (PRO 542), but more resistant to inhibition by the CD4 mab, Q4120. These data are consistent with an increased envelope affinity for CD4, although there are other potential mechanisms e.g. gp120 shedding, that could explain different sensitivities to sCD4 and PRO 542. An increased envelope affinity for CD4 could result from gp120 substitutions that that result in tighter binding to CD4, in better exposure of the CD4 binding site, or both. Certainly brain-derived envelopes are more likely to carry the N283 in the C2 CD4 binding site as reported by Dunfee et al. [35] and confirmed by our group [27]. N283 appears to confer a higher affinity for CD4 by facilitating the formation of a hydrogen bond between N283 on envelope gp120 and Q40 on CD4 [35]. We also tested envelope sensitivity to BMS-378806, a reagent reported to inhibit gp120:CD4 interactions [39,40] and gp120 conformational changes [42]. Since BMS-378806 is a small molecule, binding to gp120 will not be restricted by variable loops or glycan residues. Intriguingly, decreasing sensitivity to BMS-378806 correlated with increasing R5 macrophage-tropism. There was only minimal variation in the amino acids implicated in BMS-378806 binding which did not associate with sensitivity (not shown) [43]. The variation in BMS-378806 sensitivity must therefore be due to other mechanisms but could be explained by changes in envelope: CD4 affinities.

Protection of the CD4 binding site may be conferred by V1V2 shielding or by glycan groups [53-57]. Recently, Dunfee et al. reported that a glycosylation site at N386 may protect the proximal CD4 binding loop from neutralizing antibodies while also compromising env:CD4 interactions [36]. Curiously, N386 is a contact residue for b12 in the reported structure for b12 complexed with the HXBc2 envelope [58]. We have recently confirmed a role of N386 in protecting some envelopes from b12 (Duenas-Decamp et al. Personal communication). However, N386 contributed only modestly to the lack of macrophage infection conferred by a non-macrophage-tropic R5 envelope. Rather, we showed that residues on the N-terminal flank of the CD4 binding loop had a more significant effect on R5 macrophage-tropism and may influence the extent to which this loop is exposed (Duenas-Decamp et al. Personal communication).

Enhanced macrophage-tropism of HIV in brain tissue may result from an adaptation for infection of macrophage-lineage cells, while HIV-1 replicating in immune tissue may have adapted for replication in CD4^+ ^T-cells. However, it is unclear to what extent neutralizing antibodies in immune tissue act to modulate these different tropisms by selecting for envelopes that protect the critical envelope sites e.g. the CD4 binding site. The brain is protected by the blood brain barrier, which usually limits penetration by antibodies [37,59], although the barrier may become compromised in late disease [60,61]. We failed to show an overall significant correlation between R5 macrophage-tropism and sensitivity to any of the neutralizing mabs tested except for 2G12. The increased resistance of brain macrophage-tropic envelopes to 2G12 is not likely to be due to the presence of 2G12-like antibodies in brain tissue. Rather, 2G12 resistance may be a side effect of the evolution of variants that are less protected by glycosylation and thus lack N-linked glycosylation sites that are critical for 2G12 sensitivity. Such variants therefore may have evolved in response to the absence of neutralizing antibodies in the brain. The carbohydrate epitope for 2G12 is comprised of a cluster of α1–2 mannose residues on the outer face of gp120, which are associated with potential glycosylation sites at N295, N332, N339, N386, N392 and N448. Of these, N295 and N332 are the most important sites [46,47]. Overall, three of nine brain envelopes were resistant to 2G12, while only one of nine LN envelope were resistant. For patient NA420, all five envelopes, including those from brain and LN, lack the critical 2G12 glycosylation site at N339 [46,47] (Table 3), yet one of these envelopes (LN40) retains sensitivity to 2G12. All other envelopes retain the critical N295 and N332 residues indicating that the determinants of 2G12 sensitivity and resistance are unclear but must include other determinants in addition to these glycosylation sites.

For the CD4bs mab, b12, the lack of a correlation with macrophage-tropism is intriguing. A trend of increased b12 sensitivity for brain envelopes was observed, with all but one of the brain envelopes sensitive, while most LN-derived envelopes were resistant. The sensitivity of envelopes to b12 may also depend on whether their host patient carried antibodies that bound epitopes close to or overlapping the b12 binding site and that acted as a selective force. For example, since three non-macrophage-tropic envelopes from subject NA118 were sensitive to b12, it may be that this person did not develop such antibodies. In contrast, for patients NA20 and NA420, brain-derived envelopes were substantially more sensitive to b12 compared to LN-derived envelopes. Together, these results are consistent with selection by neutralizing antibodies that target the CD4bs or proximal epitopes in immune lymphoid tissue but not in brain. So, it seems probable that neutralizing antibodies present in immune tissue play an important role in selecting for envelopes that protect the CD4bs via variable loops, glycosylation or other mechanisms. Such envelopes may evade neutralization by antibodies, but appear to be compromised in their interactions with CD4 and limited to infection of cells that carry high amounts of CD4 e.g. CD4^+ ^T-cells. Curiously increased R5 macrophage-tropism may have resulted in increased resistance to 2G12 but increased sensitivity to b12. Thus a vaccine designed to induce both b12-like and 2G12-like neutralizing antibodies may protect against the entire range of macrophage-tropic and non-macrophage-tropic R5 viruses.

An earlier study using HIV-1 viral isolates from brain tissue suggested that their envelopes conferred a higher affinity for both CD4 and CCR5 [28]. However, our data do not support this conclusion. R5 envelopes from the brain tended to be more sensitive to CCR5 inhibitors compared to non-macrophage-tropic R5 envelopes from other sites (Figure 7), although this was not statistically significant. Thus, an increase in affinity for CD4 may reduce the requirement for a high affinity for CCR5, as suggested by Platt et al. [62]. The modulation in sensitivity to CCR5 inhibitors is clearly observed in dose dependent inhibition curves that show the majority of brain-derived envelopes from patients NA20 and NA420 are more sensitive to both TAK779 and SCH350581 (Figure 7), and the anti-CCR5 mab, 2D7 (data not shown), compared to LN-derived envelopes. NA20 LN14 is an exception that was more sensitive to CCR5 antagonists than other brain and LN-derived envelopes tested. However, LN14 carries the N283 motif present in the C2 CD4 binding site that has been associated with enhanced macrophage-tropism in the brain and increased gp120:CD4 affinity, even though this envelope was non-macrophage-tropic.

Although this study has concentrated on brain and LN envelopes, we have also included envelopes that were amplified from blood and semen. Previously, we reported that most of these additional R5 envelopes were non-macrophage tropic, although several macrophage-tropic envelopes were detected. These included C98-15 and C98-18 from the same pediatric plasma sample and the semen-derived envelopes SQ43 380.1 and 380.4. These macrophage-tropic R5 envelopes conferred increased resistance to Q4120 and enhanced sensitivity to sCD4 and PRO 542 indicating that the association of macrophage-tropism with sensitivity to reagents that interfere with envelope:CD4 interactions, holds true, regardless of envelope tissue origin.

In summary, we have studied how variation in HIV-1 R5 macrophage-tropism relates to sensitivity to neutralizing antibodies that target conserved envelope epitopes and to reagents that inhibit virus entry. We have investigated HIV-1 envelopes amplified directly from patient material without culture. Such envelopes are expected to represent those *in vivo *and have a distinct advantage over primary isolates that will have been altered by culture. Our data demonstrate considerable phenotypic variation conferred by R5 envelopes that impacts on macrophage-tropism and sensitivity to entry inhibitors including the CD4 binding site mab, b12. It is currently unclear whether this variation affects the capacity of R5 viruses to transmit. Regardless, our results strongly indicate that macrophage-tropism is modulated by changes in gp120 that predominantly impact on the CD4 binding site consistent with an increased gp120:CD4 affinity. Our results have relevance for therapies that target HIV entry and for the design of vaccines that aim to induce neutralizing antibodies.

## Methods

### Patients and HIV-1 envelopes

The subjects and envelopes described in this study have been reported previously. Subjects are summarized in Table 4. Envelopes and their tropism for macrophages are listed in Table 1. NA20 B76, NA420 LN40 and LN85 carried determinants in gp41 that compromised envelope assembly onto virus particles and conferred only low levels of infectivity. So, NA420 LN40 and LN85 envelopes used here carried gp41 sequences of NA420 B33, while NA20 B76 carried gp41 from NA20 B59. The capacity of each envelope to infect primary macrophages is described as a percent of infectivity for HeLa TZM-BL cells as reported previously [27]. Envelope sequences were PCR amplified from tissue DNA using the Expand™ High Fidelity DNA polymerase system (Roche Inc.) or KOD XL DNA polymerase (Toyobo/Novagen), both of which contain proof reading capacity. Envelopes were subcloned into pSVIIIenv via conserved Kpn I restriction enzyme sites for pseudovirion production.

**Table 4:** Details of patients studied.

Patient	Age	Status	Disease stage^a^	Neurological involvement	Samples
P3	Adult	Homosexual	B2	No	Blood Semen
P31	Adult	Homosexual	C3	No	Blood Semen
P43	Adult	Homosexual	A1	No	Blood Semen
P-1114	Neonate	MTCT^b^	A2	No	Plasma
			A2	No	Plasma
			C3	Yes	Plasma
NA118	Adult	IVDU^c^	C3	Yes	Frontal lobe, Lymph node
NA420	Adult	Heterosexual	C3	Yes	Frontal lobe, Lymph node
NA20	Adult	Hemophiliac	C3	Yes	Frontal lobe, Lymph node
NA176	Adult	IVDU	C3	Yes	Frontal lobe
NA353	Adult	IVDU	C3	Yes	Frontal lobe

^a ^Disease stage from the CDC and WHO staging system.

^b ^Mother to Child Transmission.

^c ^Intravenous Drug User.

### Neutralizing monoclonal antibodies and entry inhibitors

Human monoclonal antibodies (mabs) used here recognize conserved envelope epitopes and included b6, b12 (CD4 binding site, CD4bs) [63], 17b (CD4-induced, CD4i) [64], 2G12 (carbohydrate-dependent) [65] and gp41-specific, 2F5 [66] and 4E10 [67,68]. 2G12, 2F5 and 4E10 were obtained from the NIH AIDS Research & Reference Reagent Program and from Polymun Scientific Inc. (Austria).

Entry inhibitors included mouse anti-CD4 mab, Q4120 (specific for the N-terminal domain of CD4) [69] (The Centre for AIDS Reagents; EU Programme EVA/AVIP), soluble CD4 (derived from Chinese Hamster Ovary cells) [70], tetrameric IgG: CD4 (PRO 542 from Progenics Inc.), mouse anti-CCR5 mab, 2D7 (specific for the second extracellular loop of CCR5) (BD Biosciences Inc.), CCR5 antagonists (small organic molecules), TAK779 [71] (NIH AIDS Research & Reference Reagent Program) and SCH350581 [72] (Schering Plough Inc.), BMS-378806, a small molecule that binds a cavity deep in the gp120 cleft targeted by CD4 [43] (New England Peptide Inc.) and the gp41-specific fusion inhibitor, T20 peptide (Roche Inc.).

### Preparation and titration of envelope^+ ^pseudovirion viruses

Envelope^+ ^pSVIIIenv was cotransfected into 293T cells with env^- ^pNL43. Env^+ ^pseudovirions were harvested after 48 hours, clarified by low speed centrifugation and frozen as aliquots at -152°C. Pseudovirions were titrated on HeLa TZM-BL cells (HeLa/CD4/CCR5) cells, which carry β-galactosidase and luciferase reporter genes controlled by an HIV LTR promoter. Briefly, 500 μl HeLa TZM-BL cells (10^4 ^cells/ml) were seeded into 48-well trays 24 hours before infection with serially diluted pseudovirus. Env^+ ^pseudovirus infectivity was evaluated 48 hours after infection as focus forming units (FFU) following staining for β-galactosidase activity. Infected HeLa TZM-BL cells were washed in phosphate buffered saline, fixed in 0.5% gluteraldehyde and washed twice more in PBS. β-galactosidase substrate [26] was added to the fixed cells and infected cells stained blue. Since env^+ ^pseudovirions are only capable of a single round of replication, individual cells or small groups of divided cells were counted as foci.

### Neutralization and inhibition assays

HeLa TZM-BL cells were seeded into 96-well trays 24 hours before infection. For neutralization and inhibition assays using antibodies or inhibitors that target the HIV envelope, 200 FFU of env^+ ^pseudovirions was mixed with twofold serial dilutions of antibody or inhibitor in 50 μl. After 1 hour of incubation at 37°C, the virus/antibody mixture was added to target cells and incubated for a further 3–18 hours at 37°C. Then, the virus/antibody mixture was removed, growth medium added, and infected cells were incubated at 37°C for a total of 48 hours. Medium was then removed and 100 μl of medium without phenol red added. Cells were then fixed and solubilized by adding 100 μl of Beta-Glo (Promega Inc.). Luminescence was then read in a BioTek Clarity luminometer.

For inhibitors that target cell surface receptors (anti-CD4 Q4120, anti-CCR5 2D7, and CCR5 antagonists, TAK779, SCH350581), cells were first treated for 30 minutes with twofold serial dilutions of inhibitor or antibody in 50 μl, before adding an equal volume of env^+ ^pseudovirus containing 200 FFU. After 3–18 hours of incubation, the virus was removed. Growth medium containing the appropriate concentration of inhibitor was replenished and the infected cells were incubated for a total of 48 hours before fixing for luminescence measurements as described above.

### IC50s and correlations

IC50s and correlations were calculated using Prism 4.0c software for Macintosh. IC50s were calculated using a non-linear regression analysis. In some cases where inhibition did not completely eliminate infectivity, IC50s were estimated manually from an Excel plot. Correlations were calculated using a two-tailed, non-parametric Spearman test with 95% confidence limits.

## Competing interests

The author(s) declare that they have no competing interests.

## Authors' contributions

PJP carried out the viral infectivity and inhibition assays and contributed to the planning of experiments, overall approach and generation of the manuscript. MJD-D provided intellectual input, discussion and pertinent information from unpublished experiments. WMS provided sequence information and discussion on envelopes amplified from pediatric cases. KL provided information, discussion and details on pediatric patients. RB, CA and JB provided sequence and essential patient information as well as discussion on envelopes amplified from blood and semen from the same patients. PS and JB provided essential patient information and discussion for envelopes amplified from brain and lymph node tissue of individuals with neurological complications. JR provided 17b antibody and contributed important advice on the use of this reagent in the experiments described. DB provided the b12 antibody and contributed important advice and discussion on the experiments performed and their interpretation. PRC planned the study and wrote the manuscript with the help of PJP. All authors read and approved the final manuscript.
